# Temperature and Redox Effect on Mineral Colonization in Juan de Fuca Ridge Flank Subsurface Crustal Fluids

**DOI:** 10.3389/fmicb.2016.00396

**Published:** 2016-03-31

**Authors:** Jean-Paul M. Baquiran, Gustavo A. Ramírez, Amanda G. Haddad, Brandy M. Toner, Samuel Hulme, Charles G. Wheat, Katrina J. Edwards, Beth N. Orcutt

**Affiliations:** ^1^Department of Biological Sciences, University of Southern CaliforniaLos Angeles, CA, USA; ^2^Department of Earth Sciences, University of Southern CaliforniaLos Angeles, CA, USA; ^3^Department of Soil, Water and Climate, University of MinnesotaSt. Paul, MN, USA; ^4^Moss Landing Marine LaboratoriesMoss Landing, CA, USA; ^5^School of Fisheries and Ocean Sciences, University of Alaska FairbanksFairbanks, AK, USA; ^6^Bigelow Laboratory for Ocean SciencesEast Boothbay, ME, USA

**Keywords:** deep biosphere, geomicrobiology, oceanic crust, microbe-mineral interactions

## Abstract

To examine microbe-mineral interactions in subsurface oceanic crust, we evaluated microbial colonization on crustal minerals that were incubated in borehole fluids for 1 year at the seafloor wellhead of a crustal borehole observatory (IODP Hole U1301A, Juan de Fuca Ridge flank) as compared to an experiment that was not exposed to subsurface crustal fluids (at nearby IODP Hole U1301B). In comparison to previous studies at these same sites, this approach allowed assessment of the effects of temperature, fluid chemistry, and/or mineralogy on colonization patterns of different mineral substrates, and an opportunity to verify the approach of deploying colonization experiments at an observatory wellhead at the seafloor instead of within the borehole. The Hole U1301B deployment did not have biofilm growth, based on microscopy and DNA extraction, thereby confirming the integrity of the colonization design against bottom seawater intrusion. In contrast, the Hole U1301A deployment supported biofilms dominated by Epsilonproteobacteria (43.5% of 370 16S rRNA gene clone sequences) and Gammaproteobacteria (29.3%). Sequence analysis revealed overlap in microbial communities between different minerals incubated at the Hole U1301A wellhead, indicating that mineralogy did not separate biofilm structure within the 1-year colonization experiment. Differences in the Hole U1301A wellhead biofilm community composition relative to previous studies from within the borehole using similar mineral substrates suggest that temperature and the diffusion of dissolved oxygen through plastic components influenced the mineral colonization experiments positioned at the wellhead. This highlights the capacity of low abundance crustal fluid taxa to rapidly establish communities on diverse mineral substrates under changing environmental conditions such as from temperature and oxygen.

## Introduction

The deep subsurface biosphere represents a vast and relatively unknown environment. While this deep biosphere is estimated to comprise a significant fraction of the Earth's total biomass (Whitman et al., 1998; Kallmeyer et al., 2012), most investigations have focused on the marine sedimentary realm (Parkes et al., 2000; D'hondt et al., 2004; Biddle et al., 2006; Jørgensen and Boetius, 2007; Teske and Sørensen, 2008; Lever et al., 2010). Our understanding of microbial life within igneous oceanic crust, a potentially far larger habitat than marine sediment (Orcutt et al., 2011a,b), is slowly growing. So far, results indicate that the deep subsurface rock-hosted biosphere is different from overlying sediment in terms of the abundance and distribution of biological taxa (Thorseth et al., 2001; Cowen et al., 2003; Lysnes et al., 2004; Mason et al., 2010; Edwards et al., 2011a; Orcutt et al., 2011a; Jungbluth et al., 2013). Fluids within this highly porous and permeable crust are exchanged via advection with the overlying oceans, resulting in a flux of chemicals to and from the oceans that are significant to global geochemical budgets for some elements (Elderfield and Schultz, 1996; Wheat et al., 2003b). Such chemical exchange reactions are the foundation for chemolithotrophic microbial life (Bach and Edwards, 2003); however, factors controlling substrate colonization, bioalteration, microbial proliferation, and ecosystem establishment in crustal aquifer environments are largely unknown (Edwards et al., 2012b).

A major challenge when examining subsurface crustal microorganisms is the collection of pristine samples during drilling operations (Lever et al., 2006; Santelli et al., 2010), although there are recent examples of the possibility to detect *in situ* microbial communities with this approach (Mason et al., 2009; Lever, 2013). One method to overcome the challenge of sample recovery through drilling is through the use of CORKs (Circulation Obviation Retrofit Kits), which are subsurface observatory systems in which a borehole is cased after drilling and the hydrologic reservoir is allowed to recover to pre-drilling conditions (Davis et al., 1992; Becker and Davis, 2005; Fisher et al., 2011; Wheat et al., 2011; Edwards et al., 2012a). Long-term sampling and experimentation with CORKs is possible through a combination of fluid sampling at the seafloor wellhead, which samples fluids that originate from depth within the oceanic crust, and by the deployment of experiments within the borehole (Fisher et al., 2005; Wheat et al., 2010; Orcutt et al., 2011a; Cowen et al., 2012). Thus, CORKs offer an opportunity to access the crustal subsurface to collect fluid samples and conduct experiments.

One recent type of deep biosphere experiment enabled by CORKs is mineral colonization experiments, where sterile minerals are incubated within CORKs to examine microbe-mineral interactions. For example, flow-through osmotic colonization systems (FLOCS) enable examination of microbe-mineral interactions by slowly and continuously pumping crustal fluids over mineral substrates housed in an *in situ* growth chamber (Jannasch et al., 2004; Orcutt et al., 2010; Wheat et al., 2010). The concept of the FLOCS microbial colonization was established by incubating different minerals with fluids from a hydrothermal vent (Orcutt et al., 2010), and validation of the downhole colonization concept was achieved during a 4-year *in situ* incubation within CORKs (Orcutt et al., 2011a). Similar approaches have been used by other investigators in ocean crust (Smith et al., 2011) and in the terrestrial deep biosphere (Pedersen and Ekendahl, 1992; Pedersen et al., 2008).

**Figure 1:**
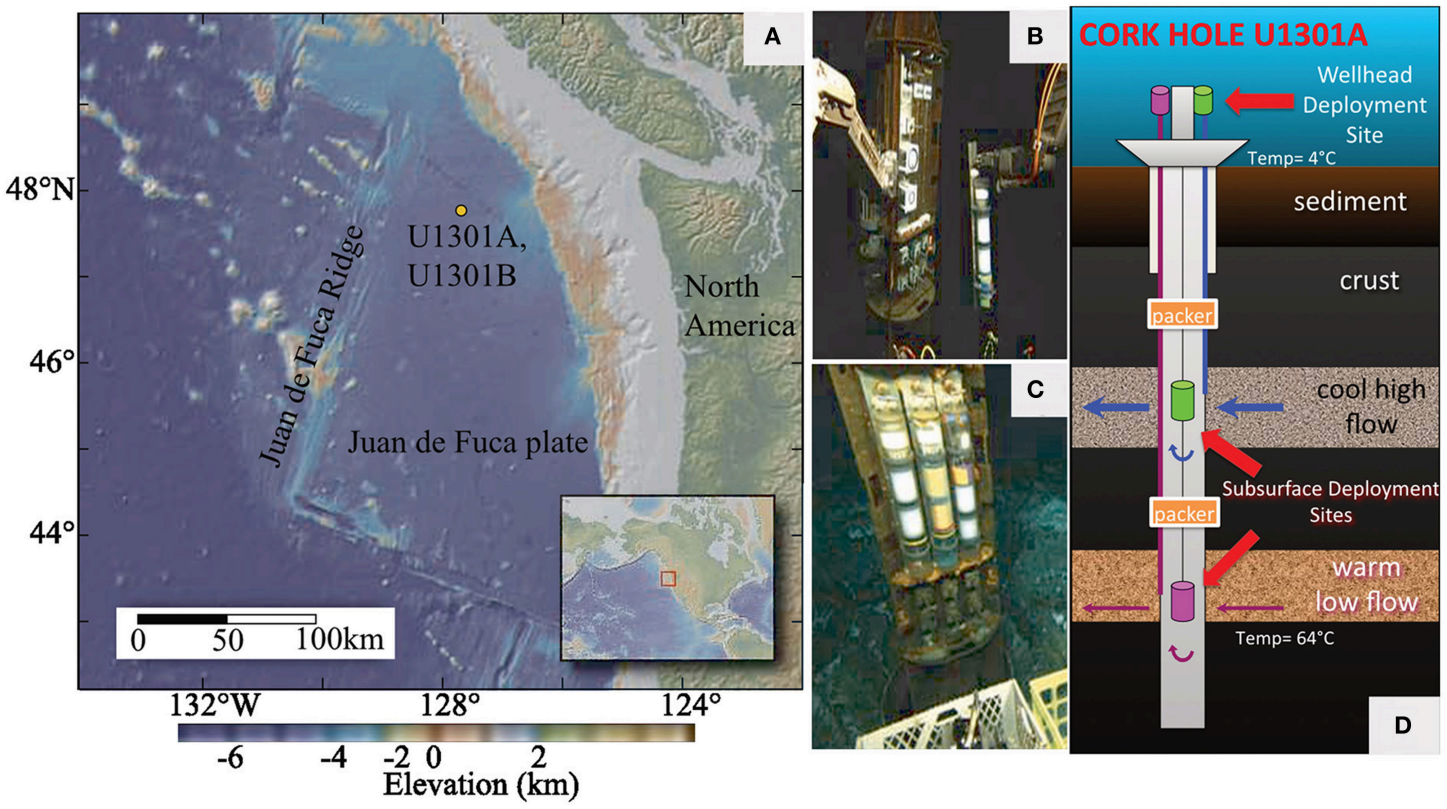
**Overview of CORK observatory experiments. (A)** Location of CORK observatories at Holes U1301A and U1301B on the Juan de Fuca Ridge flank, northeastern Pacific Ocean (modified from Fisher et al., 2011); **(B,C)** Wellhead FLOCS experiments at IODP Hole U1301A manipulated by the ROV *Jason II* robotic arms. **(D)** Hole U1301A CORK diagram highlighting wellhead seafloor deployment site (modified from Orcutt et al., 2010 and used here with permission). Photos in **(B,C)** reproduced with permission of Woods Hole Oceanographic Institution.

Initial CORK-based investigations, focused on a CORK network on the eastern flank of the Juan de Fuca Ridge (Figure 1), indicated that microbial communities hosted by the hydrothermal basaltic subsurface are distinct from other deep-sea environments, such as hydrothermal plumes, deep seawater, or seafloor-exposed basalts (Edwards et al., 2011b; Orcutt et al., 2011a,b). Moreover, microbial communities that colonize minerals are dynamic in structure and establish with relative rapidity (Orcutt et al., 2011a). Mineral composition seems to play a role in determining surface-attached microbial community structure (Orcutt et al., 2011a; Smith et al., 2011). Minerals incubated at depth within these crustal fluids were subsequently shown to enrich for microbial groups capable of iron oxidation (Smith et al., 2011). Crustal fluids collected from these CORK observatories host an order of magnitude lower cell density relative to surrounding deep seawater (Jungbluth et al., 2013), and microbial sulfate reduction coupled to organic matter oxidation is possible in these fluids (Robador et al., 2014).

The first microbe-mineral interaction studies from the Juan de Fuca Ridge flank subsurface, from experiments conducted at depth within CORKs at Integrated Ocean Drilling Program (IODP) Holes 1026B and U1301A from 2004 to 2008 (Orcutt et al., 2011a; Smith et al., 2011), offered initial insight into the microbial colonization of minerals incubated subseafloor in crustal aquifer fluids, but they were relatively limited in sample volume for deeper examination of patterns. Incubation of mineral colonization experiments at depth within a borehole has some limitations, including size restrictions to fit within the CORK, and the requirement to open the CORK to remove the instrument string at the end of the experiment, which can perturb the hydrologic system (Wheat et al., 2011). Thus, mineral colonization experiments at the CORK wellhead, where incubations are exposed to fluids rising to the seafloor from depth in the crust, were established as an alternative to the downhole incubation concept (Wheat et al., 2011; Orcutt and Edwards, 2014). While the wellhead incubation approach offers easier access for exchanging experiments and minimizes restrictions for experimental size, it was unknown if incubation at seafloor temperatures or other factors would influence the development of mineral microbial communities when compared to similar experiments deployed within a borehole.

Here, we report the first results from CORK wellhead microbial colonization experiments, which allow examination of how wellhead conditions and/or mineral compositions influence microbe-mineral interactions in the volcanic portion of the oceanic biosphere. In this experiment, FLOCS chambers were placed for 1 year at the wellhead of IODP Hole U1301A on the eastern flank of the Juan de Fuca Ridge (Figure 1). An identical deployment at the wellhead of nearby Hole U1301B served as a control, as neither subsurface fluids nor bottom seawater was introduced into the FLOCS chambers during the deployment, and there was no evidence for biofilm formation. Fluid chemical data documents that minerals deployed at Hole U1301A were exposed to fluids sourced from the igneous crustal reservoir approximately 270 m below seafloor (mbsf). 16S rRNA genes recovered from mineral substrates (i.e., basalts, sulfides, iron oxyhydroxides) in this experiment were compared to those of previous colonization experiments at depth within the borehole (Orcutt et al., 2011a) and from crustal fluids (Cowen et al., 2003; Jungbluth et al., 2013), as well as to other environmental sample sets. The wellhead colonization experiments from Hole U1301A hosted microbial communities that differed from previous analyses of this subsurface environment, reflecting psychrophilic and aerobic conditions as opposed to the thermophilic and anoxic conditions found at depth. The results highlight limitations of this experimental approach for studying microbial community structure and function of a warm and anoxic subsurface crustal system, although this wellhead incubation approach may have more success in studying cooler subsurface environments. Nevertheless, such experiments are valuable for highlighting underlying mechanisms that drive microbial colonization and succession patterns on mineral substrates.

## Materials and methods

### FLOCS chamber design, deployment, and recovery

**Figure 2:**
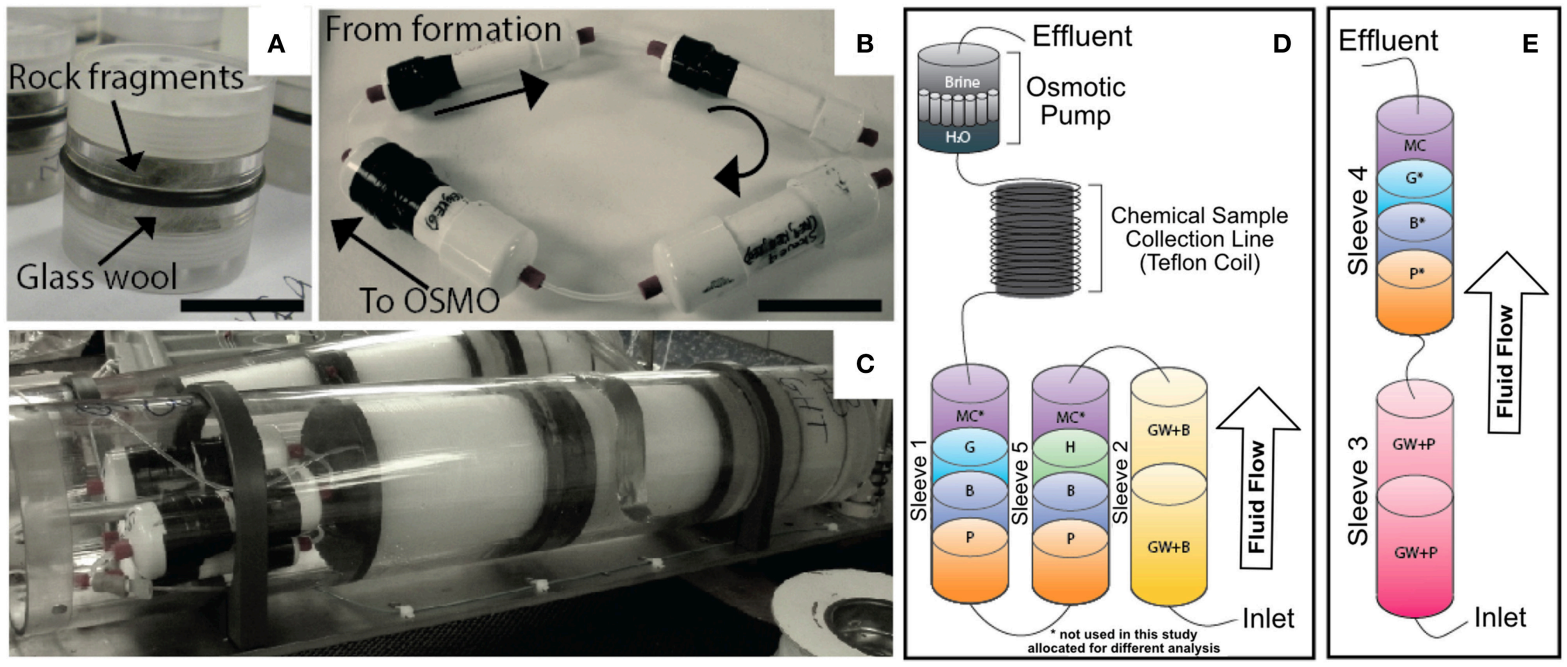
**Overview of the FLOCS experiments used in this study. (A)** Polycarbonate cassettes containing >250 μm crushed mineral substrates, with ventilated caps and o-ring seal on outside. **(B)** The FLOCS (PVC) sleeves deployed in the negative control at Hole U1301B connected in series illustrating direction of fluid flow. **(C)** The FLOCS sleeves mated to the Teflon tubing coils and osmotic pumps inside the plates to be attached to the wellhead. **(D)** Schematic of FLOCS experimental design deployed at Hole U1301A, where substrates are listed in order of fluid flow: GB, Glass wool and Basalt; P, Pyrite; B, Hole U1301B Basalt; H, Hematite; MC^*^, cm^2^ mineral chips; G, Goethite (^*^denotes substrates that were not used in this study and allocated for other analyses). **(E)** Hole U1301A passive flow (no OSMO pump) deployment labeled with identical substrate abbreviations as **(D)**.

The overall design principal of the mineral incubation devices (i.e., FLOCS) is described in detail elsewhere (Orcutt et al., 2010). The Hole U1301A deployment differed from the previous design in that the chambers were constructed of 1.5-inch schedule 40 polyvinyl chloride (PVC) plastic pipe instead of Teflon or polycarbonate (Figure 2). FLOCS chambers (sleeves) were assembled onsite, with one FLOCS chamber consisting of cassettes containing glass wool (as a means to maximize surface area for colonization) with an olivine-rich basalt from the Loihi Seamount (GW + basalt in Table 1) in Sleeve 2; cassettes of pyrite (FeS_2_) and of massive basalt previously collected from the Juan de Fuca Ridge flank subsurface at IODP Hole U1301B (“Basalt” in Table 1) in Sleeves 5 and 1; and hematite (Fe_2_O_3_) and goethite [FeO(OH)] in Sleeves 5 and 1, respectively (Figure 2). Minerals and FLOCS components were autoclaved prior to assembly, and flushed with ethanol and ultrapure deionized water after assembly, then flushed with commercially available sterile-filtered seawater (Sigma-Aldrich, product number S9148). FLOCS chambers, containing a 1:1 mixture of sterile deionized water and sterile seawater, were connected in series to a fluid sample coil (300-m-long, 1.2 mm outer diameter coil of Teflon tubing initially filled with deionized water), which was connected to an osmotic pump (Jannasch et al., 2004) with 13 semi-permeable membranes that pulled fluids through the FLOCS at ~1 ml d^−1^ at bottom seawater temperatures (Figure 2). The inlet of the FLOCS chamber was connected via gas-impermeable PEEK tubing and union fittings to a stainless steel tube that is part of the wellhead. This tubing extends to 270 mbsf into the upper volcanic crust. A separate FLOCS sleeve containing only glass wool and pyrrhotite [Fe_(1−*X*)_S (x = 0–0.2)] was linked to the same stainless steel tubing and allowed to freely vent (i.e., no osmotic pump was connected), allowing subsurface fluids to flow through the sleeve at an unknown rate due to overpressure of the subsurface fluids compared to bottom seawater (Figure 2E). The FLOCS sleeves were bundled in custom plates (Wheat et al., 2010) for deployment by the ROV *Jason II* on expedition AT15-66 (R/V *Atlanti*s; Woods Hole Oceanographic Institution) in summer 2010. The plate was connected to the right bay of the Hole U1301A CORK wellhead during dive J2-498.

**Table 1:** **Abundance and taxonomic classification of 16S rRNA gene sequence clone groups (focusing on classes of Proteobacteria) from each mineral from Hole U1301A, with an operational taxonomic unit (OTU) defined as 97% or higher sequence similarity**.

**Group**	**OTU ID**	**% of clones**	**# clones**	**GW and Basalt**	**Basalt**	**GW and Pyrrhotite**	**Pyrite**	**Hematite**	**Goethite**	**Closest relative—environment (Accession #)**	**%**	**References**
Alpha	21	6.8	25	25	–	–	–	–	–	*Roseobacter*—Lost City hydrothermal (DQ270648)	97	Brazelton et al., 2006
	18	2.4	9	3	–	1	4	1	1	*Thalassospira*—ridge flank crustal fluids (DQ490020)	99	Huber et al., 2006
	3	2.2	8	–	3	–	–	5	–	*Parvibaculum*—Deepwater Horizon spill (JN018430)	99	Redmond and Valentine, 2012
	23	0.3	1	–	–	–	1	–	–	*Parvibaculum*—borehole crustal fluid (GQ903365)	96	Nigro et al., 2012
	12	1.1	4	–	2	1	1	–	–	Alphaproteobacteria—ocean water (JN717169)	96	Luan et al., unpublished
	9	0.3	1	–	1	–	–	–	–	Alphaproteobacteria—deep ocean (HM798889)	96	Eloe et al., 2011
Beta	15	1.9	7	6	1	–	–	–	–	*Deftia sp*. (NR_074626)	99	Lucas et al., unpublished
	7	0.8	3	2	1	–	–	–	–	*Ralstonia picketti* (CP001645)	99	Lucas et al., unpublished
	14	0.8	3	1	1	1	–	–	–	*Ralstonia solanacearum* (EF016365)	98	Albequrque et al., unpublished
Gamma	1	12.7	47	5	19	4	8	8	3	*Shewanella*—deep-sea surface sediment (AM997576)	98	Schauer et al., 2010
	2	11.1	41	–	21	2	–	18	–	*Neptunamonas*—deep-sea whale fall (AY549004)	99	Goffredi et al., 2005
	8	4.1	15	1	11	–	–	3	–	*Colwellia psychrerythraea 34H* (CP000083)	99	Methé et al., 2005
	16	1.4	5	4	–	1	–	–	–	deep-sea *Pseudomonas sp*. (AM410618)	99	Zhang et al., unpublished
Epsilon	4	27.3	101	–	8	11	34	20	28	*Arcobacter*—Black sea shelf sediment (AJ271654)	95	Thamdrup et al., 2000
	11	12.4	46	–	2	–	17	–	27	*Arcobacter sp*. From North Sea (AF235110)	95	Eilers et al., 2000
	13	3.5	13	–	1	–	1	11	–	Black sea shelf sediment *Arcobacter sp*. (AJ271654)	96	Thamdrup et al., 2000
	5	0.3	1	–	1	–	–	–	–	*Sulfurimonas*—hydrothermal vent (FN554112)	97	Schauer et al., 2011
Other	17	0.3	1	–	–	1	–	–	–	*Mariprofundus*—hydrothermal sulfides (JQ287464)	99	Sylvan et al., 2012
	6	4.9	18	–	6	–	5	–	7	Flavobacteraceae—Japan trench cold seep (AB015261)	99	Li et al., 1999
	19	3.0	11	1	–	1	7	1	1	Bacteriodetes—hydrothermal sediment (AF419687)	98	Teske et al., 2002
	10	1.1	4	1	1	–	–	2	–	*Lutibacter*—Arctic Ocean sediment (EU287256)	99	Li et al., 2009
	22	0.3	1	1	–	–	–	–	–	*Lactobacillus perolens* (NR_029360)	95	Buck et al., unpublished
	20	0.8	3	–	–	–	2	–	1	Unclassified ridge flank crustal fluid (AY704403)	97	Huber et al., 2006
	24	0.3	1	–	–	–	1	–	–	Unclassified hydrothermal sulfide (JQ287080)	95	Sylvan et al., 2012
	25	0.3	1	–	–	–	1	–	–	Unclassified cold marine seep (FJ814754)	94	Buck et al., unpublished
		Total	370	50	79	23	82	69	67	

*Closest environmental sequence relatives determined are shown with Genbank accession number and percent sequence similarity*.

In parallel, a replicate set of FLOCS chambers were attached to the wellhead at Hole U1301B, 300 m from Hole U1301A. Here the stainless steel umbilicals that reach to the volcanic reservoir at depth are obstructed, preventing the exchange of borehole fluids with the FLOCS during the course of the deployment. Moreover, during deployment the tubing connecting the pump to the Teflon coil on the outflow of the FLOCS chambers was accidentally disconnected, preventing the pumping of any fluids into the FLOCS chamber and coil. Thus, this deployment was used as a control that (1) verifies the absence of inadvertent contamination during FLOCS assembly/deployment/collection, (2) provides confirmation of the sterility of filtered seawater used in the FLOCS sleeves during deployment, and (3) confirms the physical integrity of colonization design against background seawater intrusion. Thus, Hole U1301B results verify that observations on the Hole U1301A materials resulted from exposure to volcanic reservoir fluids.

The FLOCS experiments were recovered during ROV *Jason II* dive J2-566 on cruise AT18-07 (R/V *Atlantis*) in summer 2011. Immediately after recovery, FLOCS sleeves were disassembled with ethanol and flame-sterilized tools, and samples were stored for shore-based DNA and microscopy analysis at −80°C.

### Chemical analysis of fluids exiting the FLOCS experiments

In principle, during the deployment of the Hole U1301A FLOCS experiment, fluids sourced from the subsurface mixed with the FLOCS contents, eventually flushing out the initial chamber fluids and replacing it with subsurface-origin fluids. These mixed fluids would then be continuously collected in the sampling coil attached to the outflow end of the FLOCS experiments (Figure 2D). Thus, a record of the history of dissolved fluid chemistry in the FLOCS experiment can be obtained by sectioning the Teflon tubing attached downstream of the FLOCS, as described in detail elsewhere (Wheat et al., 2010). Here, the Teflon coils attached to the Hole U1301A and U1301B FLOCS were separated shipboard in 1-m intervals to collect the FLOCS effluent for bulk chemical analyses; these subsamples were then acidified according to established protocols (Wheat et al., 2011). Since the fluids were not preserved *in situ* with acid to dissolve metal-containing compounds that precipitate upon exposure to oxic conditions (i.e., the Teflon tubing allows penetration of oxygen; Wheat et al., 2010), particle formation in the sampling coil may have skewed the time-series of redox sensitive elements. Major and minor ion concentrations were measured on select samples using inductively coupled plasma mass spectrometry (ICP-MS) following established procedures (Wheat et al., 2010).

### DNA extraction, 16S rRNA gene clone library construction, and sequence analysis

DNA was extracted from roughly 2 g of crushed mineral chips from each substrate using the FastDNA® Spin Kit for Soil (MP Biomedicals) according to the manufacturer's protocols. DNA was quantified using a Qubit 2.0 fluorometer with HS DNA reagent kit (Invitrogen). Due to low biomass yields (i.e., DNA concentrations below detection limit, no PCR bands directly from DNA extracts), environmental DNA was amplified using the illustra GenomiPhi V2 DNA Amplification Kit (GE Healthcare) according to manufacturer's protocols. The amplified DNA was then used as a template for PCR amplification of the full-length 16S rRNA gene using the universal primers B27F and U1492R (Weisburg et al., 1991). PCR reaction parameters are found elsewhere (Baquiran et al., 2012). The resulting PCR products were purified using the QIAquick PCR Purification Kit (Qiagen), then ligated and cloned using the TOPO TA Cloning® Kit (Invitrogen). Clone libraries from each substrate were constructed and sequenced by Beckman Coulter Genomics. Sequences were assembled using *Geneious R6* and then aligned with the *MAFFT* alignment program (Desantis et al., 2006; Schloss, 2009; Drummond et al., 2012). Chimeric sequences were removed using *Bellerophon*, phylogenetic trees were constructed using *PhyML 3.0* and community dendograms were made with *mothur* version 1.35.1 (Guindon and Gascuel, 2003; Huber et al., 2004; Schloss, 2009; Drummond et al., 2012). Classifications of 16S rRNA gene sequences were performed with the *Silva* ribosomal RNA database using the *SINA* v1.2.11 aligner and the NCBI database using the BLAST algorithm (Altschul et al., 1997; Schloss, 2009; Pruesse et al., 2012). OTUs were arbitrarily numbered according to the order of detection from clone libraries. Additional sequences used for phylogenetic and community analyses were accessed from GenBank. Sequence data were submitted to GenBank under accession numbers KC682104–KC682473.

To examine the microbial community structure of the Hole U1301A wellhead minerals to previous examinations of mineral biofilm community composition, we calculated membership-based pairwise similarity matrix indices between the Hole U1301A wellhead FLOCS clone sequences with clone sequences from inactive seafloor sulfides from the East Pacific Rise (Sylvan et al., 2012), a mineral biofilm collected from a CORK at Hole 896A on the Costa Rica Rift (Nigro et al., 2012), East Pacific Rise seafloor basalts (Santelli et al., 2008), a 3-year time series of crustal fluids from Hole U1301A (Jungbluth et al., 2013), and a mineral colonization experiment incubated at depth in Hole U1301A at 64°C (Orcutt et al., 2011a). Pairwise community comparisons for shared community composition and species richness where performed using the following *mothur* calculators: J_class_, J_est_, Kulczynski, Lennon and Anderberg (Schloss, 2009), and cladograms from each metric were compared to examine trends.

### Scanning electron microscopy

To assess for biofilms on the control incubation minerals from Hole U1301B, scanning electron microscopy was conducted on select mineral substrates (basalt and pyrite) from the experiment, following approaches published previously (Orcutt et al., 2011a). Mineral coupons were thawed to room temperature and mounted on carbon tape and coated with approximately 30 μm of graphite to reduce charging. Images were obtained using a JSM 6610 low-vacuum SEM (JEOL USA, Inc.) at 5 kV and a working distance of 10 mm with a point-to-point resolution of 3 nm in the Center for Electron Microscopy and Microanalysis (University of Southern California). Similar analyses were not possible on Hole U1301A incubated minerals due to consuming all available material for DNA-based analyses.

## Results

### Fluid composition

**Figure 3:**
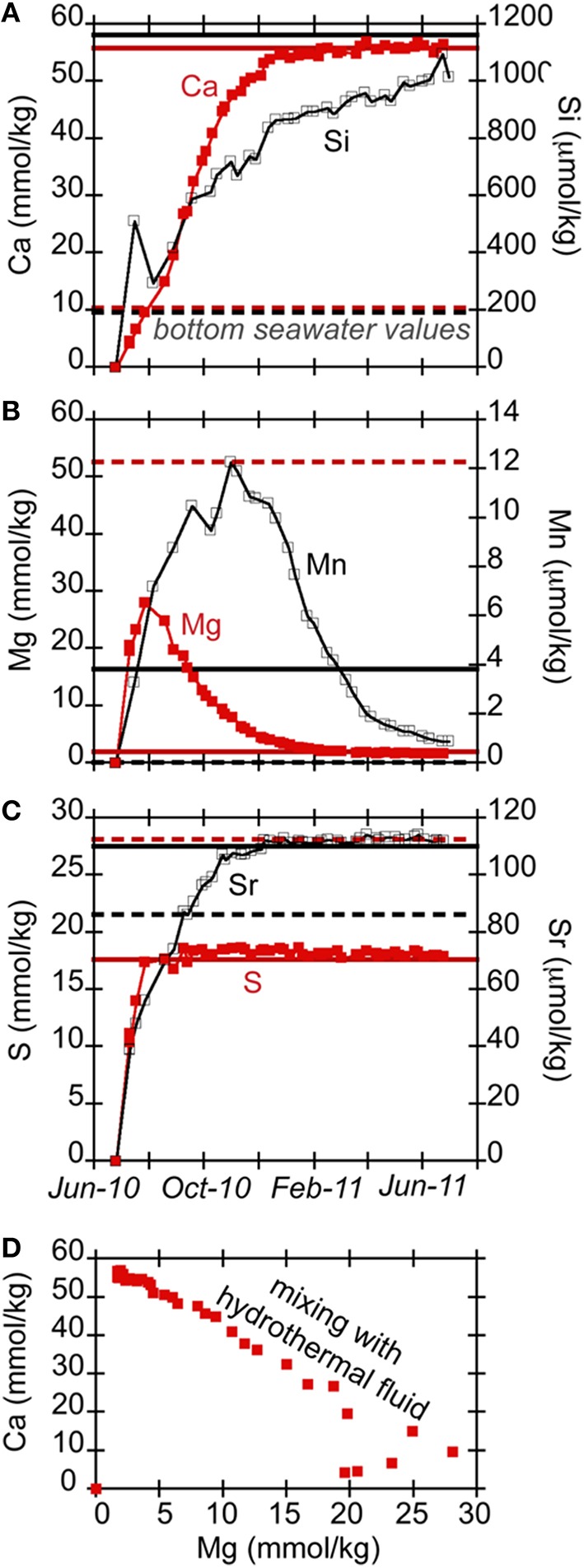
**Ion concentrations [(A) Ca (red filled squares) and Si (black open circles); (B) Mg (red filled squares) and Mn (black open circles); (C) S (red filled squares) and Sr (black open circles)] in Hole U1301A FLOCS experiment plotted as a function of time, showing the chemical evolution of the FLOCS experiment from initial conditions when filled with diluted sterile seawater to an eventual replacement with crustal fluids sourced from the volcanic crust**. Dashed and solid horizontal lines in **(A–C)** indicate concentrations of ions in bottom seawater and Hole U1301A crustal fluids, respectively, as presented in Wheat et al. (2010). **(D)** presents the Ca vs. Mg concentrations for these samples, demonstrating the mixing of different end member fluids.

Previous long-term chemical records of fluids at Hole U1301A indicate upper basement fluids (~300 mbsf) are typically warm (64°C) and anoxic yet relatively high in sulfate and calcium (Wheat et al., 2010; Lin et al., 2012), with the elevated calcium indicative of hydrothermal fluid-rock interactions. In this study, the fluids that bathed the rock substrates evolved over time due to flushing out of the initial chamber contents (a mixture of sterile seawater and deionized water) with the deep-sourced crustal fluids (Figure 3). Notably, calcium concentrations increased monotonically from the concentration of distilled water that initially filled the sample tubing before deployment and reaches an asymptote concentration near 56 mmol kg^−1^ after 5 months of deployment (Figure 3A), which agrees with regional Ca concentrations in upper basement (Wheat et al., 2010). Concentrations of dissolved silica (Si) also increased throughout the experiment, with a maximum of 1160 μmol kg^−1^. This is about the same concentration of borehole fluids 3 years after the initial drilling (Wheat and Mottl, 2000; Wheat et al., 2003a, 2010). Concentrations of magnesium changed with time to reach 1.7 mmol kg^−1^ by the end of the experiment (Figure 3B), approaching the crustal fluid composition of 1.9 mmol kg^−1^ (Wheat et al., 2010). Sulfate and strontium concentrations increased to 18 mmol kg^−1^ and 110 μmol kg^−1^, respectively (Figure 3C), consistent with earlier results (Wheat et al., 2010). Measured dissolved iron concentrations are not reliable because precipitation in the sample coil is possible during the deployment, since the FLOCS effluent was not preserved with acid during collection.

Manganese initially increased within the first few months to over 12 μmol kg^−1^ (Figure 3B), then decreased with time to approach low values (<0.001 μmol kg^−1^) observed in both bottom seawater and borehole fluids (Wheat et al., 2010). Possible oxidants of Mn, such as oxygen and nitrate, are missing from the source hydrothermal fluids (Mottl et al., 1998), which led us to explore the possibility for oxygen intrusion into the experiment. The PVC plastic used in the construction of the FLOCS is permeable to bottom seawater oxygen. Assuming a 5% decrease per degree Celsius change in the oxygen permeability of PVC at standard state, and given the surface area for each FLOCS chamber, we calculate that roughly 350 nanomole of oxygen per day could penetrate into a PVC FLOCS chamber at the seafloor (at a maximum, as this rate would decrease with a decreasing gradient in oxygen across the plastic). Given the variable FLOCS chamber volume occupied by fluid (as opposed to solid rocks) and the pumping speed of the attached OsmoSampler pump, we estimate that it would take roughly 4 weeks for the oxygen concentration in the FLOCS to reach background seawater oxygen concentrations (~100 micromolar; Brian Glazer, personal communication) if no oxygen removal processes occurred within the FLOCS.

As expected, the Teflon coil connected to the FLOCS experiment at Hole U1301B contained distilled water, as determined by initial refractometer measurements of the fluids (data not shown), indicating that no fluids (seawater or crustal) had passed through this experiment. Thus, further chemical analyses were not performed.

### Bacterial diversity and membership analyses

**Figure 4:**
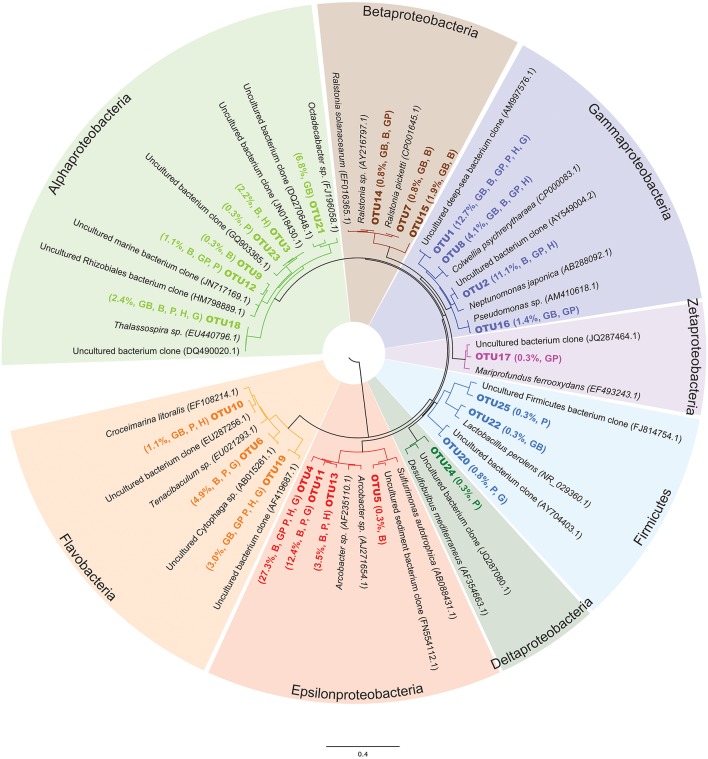
**Bacterial 16S rRNA gene neighbor-joining phylogenetic analysis of representative OTU sequences compared to close environmental sequence relatives from the Silva database**. OTUs in this study are labeled in bold. Relative abundance of the OTU in the entire sequence library (i.e., percent out of 370 clones) and abbreviation of sample type that the OTU was found in listed in parentheses after each OTU name, using the same sample code as listed in Figure 2.

To evaluate microbial colonization patterns on the different mineral substrates, environmental DNA was extracted from the samples, and then 16S rRNA genes of Bacteria were amplified, cloned and sequenced. Original DNA concentrations were below detection limit, so DNA was amplified with Phi29 Polymerase, then the 16S rRNA gene was amplified with bacterial specific primers via PCR. Hole U1301A minerals produced visible bands on an agarose gel, and the product was used for further cloning, whereas the Hole U1301B minerals did not generate visibly amplified DNA and no further cloning attempts were made. In total, 370 nearly full-length 16S rRNA gene sequences were obtained from Hole U1301A minerals, ranging from 23 to 82 clones per substrate (Table 1). At the 97% or greater sequence similarity operational taxonomic unit (OTU) classification level, there were a total of 25 unique OTUs from the 370 16S rRNA gene sequences (Table 1, Figure 4).

An overview of the OTU diversity and abundance from the Hole U1301A minerals reveals that sequences grouping within the Epsilonproteobacteria were the most abundant in the clone libraries (161 of 370 sequences; Table 1, Figure 4). OTU4 was the most prevalent clone sequence in the library (27.3% of all clones), and it was detected in all samples except the glass wool + basalt sample (Table 1). OTU4 is most closely related to the *Arcobacter* genus (Table 1, Figure 4). OTU11, the third most abundant clone type, is also closely related to species from the *Arcobacter* genus. This phylotype was abundant in the pyrite and goethite samples but absent or low count in clones from other samples (Table 1). OTU13 was also *Arcobacter* related and found predominantly on hematite (Table 1). The only other Epsilonproteobacteria-related OTU was related to the *Sulfurimonas* genus and was observed on the basalt sample. In summary, *Arcobacter sp*. related clones were the dominant group in the Hole U1301A FLOCS (43.2% of all clones), and these were abundant on the iron oxides (hematite, goethite) and iron sulfides (pyrite, pyrrhotite) but less abundant on the basalt samples (Table 1).

Gammaproteobacteria related sequences were the second most abundant clone library group (108 clones, 29.3% of all clones; Table 1, Figure 4). OTU1 was classified within the *Shewanella* genus and was the only clone type present in every substrate of this experiment. OTU2, represented by 41 clones with high relative abundances in hematite and basalt, also groups with Gammaproteobacteria close to *Neptunamonas*-related spp. from deep-sea whale falls. OTU8 is most closely related to the *Colwellia* genus and was most abundant on basalt. Lastly, *Pseudomonas*-related OTU16 in the Gammaproteobacteria was only amplified from the glass wool + mineral substrates. Collectively, four different Gammaproteobacteria phylotypes were observed with high relative abundance in basalt mineral substrates and lower abundance on the sulfides.

Of the remaining sequence groups, 48 clones were classified as Alphaproteobacteria, with the majority of these (25 clones) being closely related to *Roseobacter* from hydrothermal fluids and found exclusively on the glass wool + basalt sample (Table 1, Figure 4). Other Alphaproteobacteria-related sequences were found on multiple sample types but in relatively low abundance. Three low abundance Betaproteobacteria related phylotypes were observed on the glass wool and basalt substrates and grouped most closely with the *Ralstonia* genus. One clone from pyrite, OTU24, grouped with unclassified Deltaproteobacteria from deep-sea hydrothermal sulfides. One *Mariprofundus*-related sequence within the Zetaproteobacteria was documented from the glass wool + pyrrhotite sample. Two Flavobacteria-related phylotypes were amplified from each of the substrates at low relative abundances.

**Figure 5:**
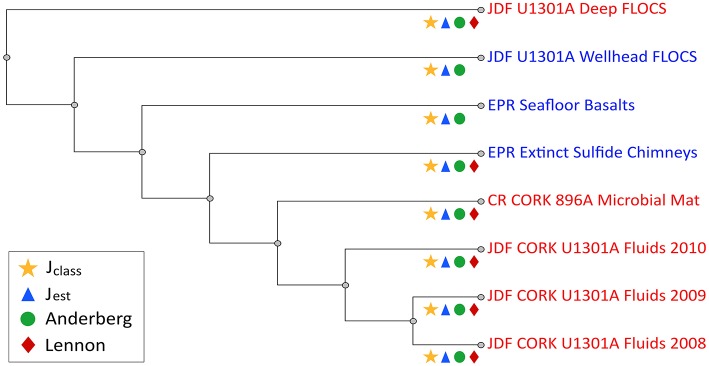
**Kulczynski cladogram summarizing results of four other pairwise similarities in community membership calculators (J_**class**_, J_**est**_, Anderberg, and Lennon) comparing combined OTUs to various environmental sites**. Site names, depicted as cladogram leaves, are color coded in red and blue to indicate warm (58–64°C; Hole U1301A Deep, Hole U1301 2008-2010 Crustal fluids, Costa Rica Hole 896A CORK Microbial Mat) and cold (seafloor, ~4°C; Hole U1301A Wellhead FLOCS, East Pacific Rise seafloor Basalts, Extinct Sulfide Chimney) incubation environments, respectively. Symbols on each branch indicate which community membership calculators supported this branching order, as shown in legend.

Community similarity based on sequence membership, as evaluated by several different indices, consistently clustered Hole U1301A wellhead FLOCS mineral microbial communities as branching between Hole U1301A borehole-incubated minerals and seafloor-exposed minerals (basalts and sulfides), with Hole U1301A fluids and a mineral biofilm from Hole 896A grouping more distantly (Figure 5).

### Mineral alteration products

**Figure 6:**
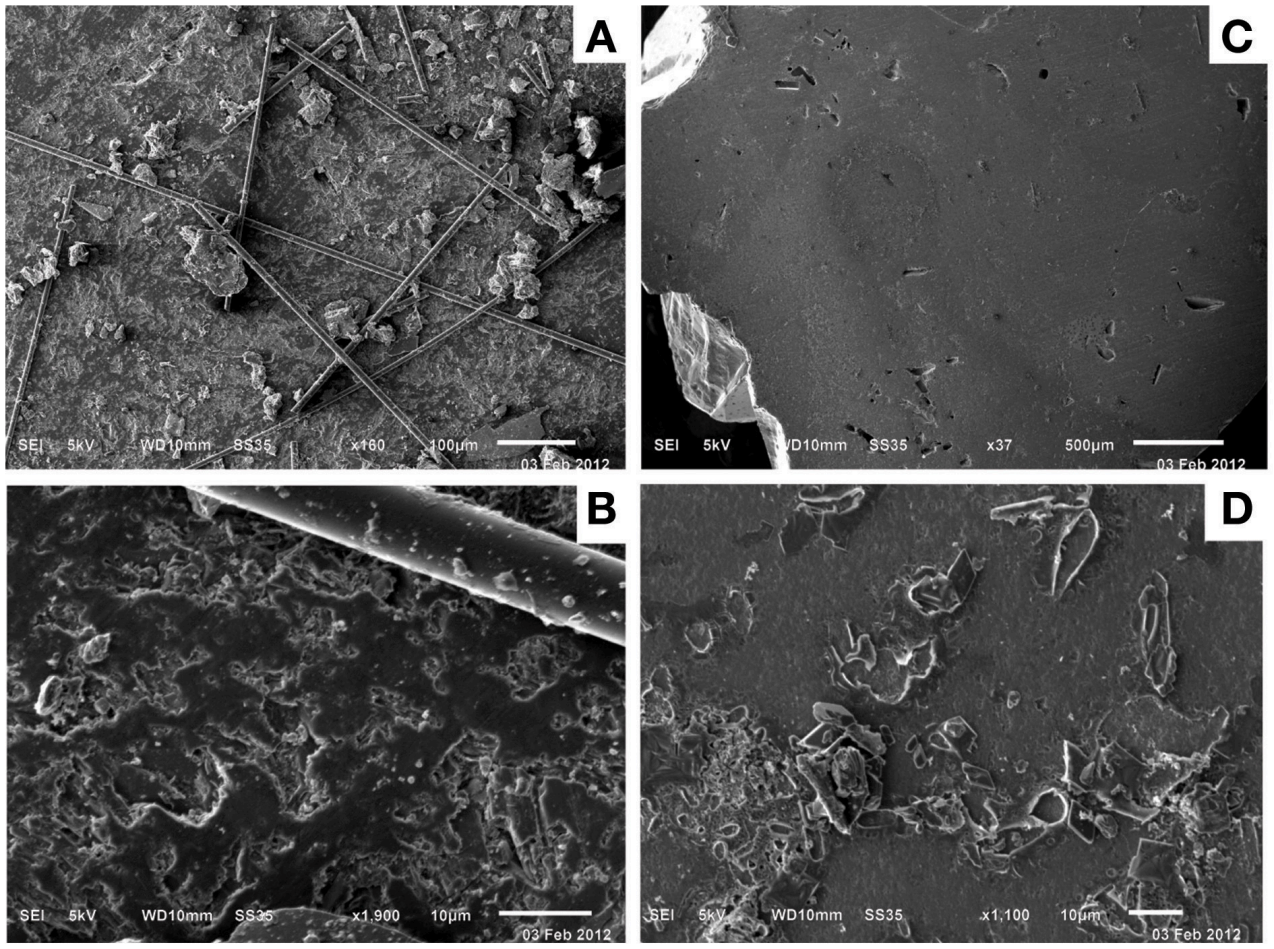
**Scanning electron microscopy of control Hole U1301B FLOCS minerals revealed limited evidence for biofilms or secondary mineral alteration**. Basalt mineral coupon SEM **(A,B)** revealed thin filaments, most likely fragments of glass wool from the FLOCS experiment. Pyrite mineral coupon SEM **(C,D)** revealed a paucity of particles or alteration. Scale bars lengths are 100 **(A)**, 10 **(B)**, 500 **(C)**, and 10 μm **(D)**.

To examine whether microbial biofilms formed on the surface of minerals incubated in the Hole U1301B FLOCS, mineral coupons were examined by SEM. No alteration products or microbial biofilms were observed on Hole U1301B mineral coupons via SEM (Figure 6). SEM revealed thin filaments on the basalt coupon. These are most likely fragments of the glass wool from the FLOCS that the mineral chips were embedded within. The basalt coupon had scant amorphous particulate matter (Figures 6A,B). The pyrite coupon was devoid of secondary minerals on the surface (Figures 6C,D).

## Discussion

In the quest to study microbial life supported by the deep crustal biosphere—a major focus of the international ocean drilling program (Iodp 2003-2013 Initial Science Plan, 2001)—this experiment set out to examine the utility of deploying mineral colonization experiments at the wellheads of crustal subsurface borehole observatories (i.e., CORKs) as an alternative to downhole deployments (Figure 2). The motivation for such deployments is the relative ease of replacing wellhead experiments using ROVs or submersibles without opening the borehole to remove experiments, which can disturb the system. To this end, mineral colonization experiments were deployed at the wellheads of two CORKs on the Juan de Fuca Ridge flank that permit access to anoxic, warm (64°C) crustal fluids (Figure 1). Here, we show that microbial communities sourced from subsurface crustal fluids at Hole U1301A can colonize minerals incubated at seafloor CORK wellheads within a 1-year period, and that the wellhead incubation concept is robust against the intrusion of bottom seawater (based on a control experiment at Hole U1301B). Colonization occurs despite crustal fluids from this region containing approximately an order of magnitude lower cell densities compared to bottom seawater (Jungbluth et al., 2013), and relatively slow fluid flushing rates of the experiments (~1 ml d^−1^), both of which exacerbate low biomass conditions.

The results reveal that temperature and physico-chemical conditions at the wellhead have a strong influence on the structure of microbial communities that develop on mineral surfaces. For instance, wellhead-incubated minerals bathed in crustal subsurface fluids at bottom seawater temperature hosted microbial communities dominated by Epsilonproteobacteria and Gammaproteobacteria related to sulfur and iron oxidizing bacteria (Figures 4, 5), whereas anoxic and warm minerals and fluids from depth in the crust at this location harbor microbial communities with significantly different membership and structures dominated by thermophilic, spore-forming Firmicutes and sulfate-reducing bacteria (Orcutt et al., 2011a; Smith et al., 2011; Jungbluth et al., 2013). The wellhead-incubated minerals hosted microbial communities reminiscent of seafloor-exposed rocks that are bathed in cold oxygen-rich seawater, suggesting cold and oxic conditions in the wellhead FLOCS experiment structured the microbial community that colonized the minerals. Thus, although these experiments have limited utility for the intended purpose of understanding the dominant microbe-mineral interactions in the warm and anoxic subsurface at Juan de Fuca, they do have utility for examining the adaptive capacity of subseafloor microbial communities under changing environmental conditions (for example, where hydrothermal fluids mix with oxic seawater near hydrothermal vents). Moreover, temperature and physical-chemical influences may not be an issue when conducting wellhead experiments in cool and oxic ridge flank systems, such as at similar CORKs on the flanks of the Mid-Atlantic Ridge (Edwards et al., 2012b; Orcutt et al., 2013a). Hence, these results highlight the capacity for adaptation for subsurface crustal microbial communities to respond to changing environmental conditions, and will guide future experiments to examine microbe-mineral interactions in the subsurface.

### Development of wellhead FLOCS chemical conditions in comparison to the borehole environment

Collectively, the chemical data from the Hole U1301A FLOCS experiment indicate simple three-component mixing upon which reactions are exposed (Figure 3D). Given a sample coil that is initially filled with distilled water and FLOCS chambers that are filled with a mixture of sterile-filtered seawater and distilled water, the initial fluids recovered in the sample coil reflect a mixture of these two solutions, consistent with the initial increase in Mg concentrations. As the hydrothermal fluid is pulled into the FLOCS, mixing occurs between the initial FLOCS fluid and this hydrothermal fluid, also producing a linear (conservative) mixing trend (e.g., Ca vs. Mg). This mixing continues until the fluid in the FLOCS is similar to the composition of the crustal fluid (e.g., asymptote for Ca and Mg vs. time). Notably, the major ion chemistry confirms that the Hole U1301A FLOCS were exposed to crustal fluids and not bottom seawater.

In contrast, Mn data do not follow such trends (Figure 3B), suggestive of reaction within the FLOCS. Mn is leached from rock substrates, as has been observed previously (Orcutt et al., 2010), either from abiotic or biotic processes. With time, Mn values decrease to concentrations below the basement fluid concentration, consistent with Mn removed from solution. A simple model of oxygen diffusion across the PVC plastic used in the experiment suggests that the interior of the FLOCS experiment became oxic within a few weeks, despite the slow pumping of initially anoxic crustal fluids through the experiment. Thus, use of PVC FLOCS chambers for wellhead mineral colonization experiments compromises an essential chemical condition for studying the anoxic Juan de Fuca Ridge flank environment. Therefore, the structure of mineral-attached microbial communities in the wellhead experiments cannot be extrapolated to the subsurface environment at this site. Nonetheless, the experiments do allow assessment of biological adaption to changing environmental conditions (i.e., warm and anoxic vs. cool and oxic). To match subsurface conditions, future wellhead colonization experiments at this site should be placed within the open borehole which vents anoxic crustal fluids at ~62°C (Wheat et al., 2004, 2010; Fisher et al., 2012), or they should be constructed of gas-impermeable materials, such as the Teflon-lined stainless steel devices used in the terrestrial subsurface (Pedersen, 2012).

### Comparison of microbial communities on wellhead, borehole, and seafloor-exposed minerals

Comparison of microbial community structure based on amplified DNA can be challenging due to possible amplification biases. In addition to biases associated with any PCR primer set (Suzuki and Giovannoni, 1996), bulk genomic DNA amplification prior to 16S rRNA gene PCR may introduce additional representational inconsistencies. Because the amount of original template DNA prior to multiple displacement amplification was very low in our experiments (<1 ng ml^−1^) with a relatively diverse community, the representational biases are assumed to be minimal (Suzuki and Giovannoni, 1996; Binga et al., 2008). Thus, we assume that the abundance of clones generated from different minerals can be compared to assess relative abundance.

The combined effects of low temperature and aerobic conditions influenced the wellhead mineral colonization microbial community structure relative to similar mineral substrates incubated at depth under warm and strictly anoxic conditions (Figure 5). Previous experiments showed that incubation of mineral substrates in anoxic crustal fluids at 64°C resulted in microbial communities indicative of anaerobic, thermophilic, and spore-forming organisms (Orcutt et al., 2011a), similar to microbial communities observed in the anoxic fluids sampled from this environment (Jungbluth et al., 2013; Robador et al., 2014). In contrast, microbial communities established on similar minerals incubated in the same crustal fluid source but under low temperature and oxic conditions at the wellhead were dominated by members reflecting aerobic, microaerophilic, or facultatively anaerobic lifestyles (Table 1, Figure 4). The most prevalently recovered 16S rRNA gene sequences were classified as *Arcobacter* spp. (Table 1), members of the Epsilonproteobacteria known for sulfur oxidation metabolic capacity. While Epsilonproteobacteria-related sequences have not previously been found in the anoxic borehole environment of the Juan de Fuca Ridge flank subsurface (Cowen et al., 2003; Orcutt et al., 2011a; Smith et al., 2011; Jungbluth et al., 2013), they have been detected in the mixed hydrothermal fluids that vent from springs on Baby Bare outcrop, about 50 km to the south along the same crustal fluid flow path as Hole U1301A (Huber et al., 2006), suggesting that these low abundance members (Sogin et al., 2006) of the warm and anoxic crustal subsurface can proliferate under suitable cooler and/or oxic environmental conditions. Similar Epsilonproteobacteria have been detected on seafloor-exposed basalt and sulfide minerals near other hydrothermal systems (Orcutt et al., 2010; Sylvan et al., 2012; Toner et al., 2013), suggesting a widespread distribution of mineral-dependent Epsilonproteobacteria.

At a broader level, examining the wellhead FLOCS microbial community structure as a whole in comparison to other environmental studies reveals the blending of the wellhead FLOCS microbial community between subsurface, warm, anoxic conditions with seafloor, cool, oxic conditions (Figure 5). Community membership calculators predict intra-community similarities for Hole U1301A crustal fluids, while all other communities are independent branches with no additional significant intra-community similarities but defined inter-community membership patterns. A temperature structure exists; however, downhole FLOCS incubations appear to be an exception. Crustal fluids (64°C) sampled from Hole U1301A during three consecutive years are most similar to a microbial mat collected from the exterior of a sub seafloor observatory port venting 58°C fluids into the surrounding bottom water (Hole 896A CORK). All 4°C environment communities (i.e., extinct sulfide chimneys, seafloor basalts, and wellhead Hole U1301A FLOCS) consistently group together as similar in inter-community membership with deep independent branching relative to the 58°C venting CORK fluid biofilm and 64°C crustal fluid communities. All community calculators used here placed Hole U1301A FLOCS incubated downhole as the outgroup, implying that, in addition to temperature, other variables, such as the lack or presence of dissolved oxygen, must be considered to more accurately explain community membership similarity observations.

Recent studies have suggested that the deep oceanic crustal biosphere has a dynamic nature, where the structure of microbial communities can change on a relatively rapid timescale, reflecting changes in chemical and thermal conditions (Orcutt et al., 2011a; Jungbluth et al., 2013). One unknown in that phenomenon is the rate and extent at which microbes can grow and respond to physico-chemical changes in substrate-attached dark ecosystems. Temperature differences probably result in drastically different selection criteria for enzymes suited for temperature-specific biochemical activity and associated taxon recruitment and/or subsequent biological succession shifts. Here, analyses of mineral colonization FLOCS experiments deployed at a CORK wellhead, sampling fluids sourced from 270 mbsf, indicate that relatively diverse assemblages of bacteria sourced from sub seafloor aquifer fluids can colonize sterile mineral surfaces at both 64°C under anoxia (Orcutt et al., 2011a) and at 4°C under aerobic conditions (this study) within a year. Combined, these observations highlight the dynamic and environmentally responsive nature of microbial assemblages endogenous to crustal fluids at Hole U1301A. The colonization rapidity and thermal/redox selectivity suggests that subseafloor aquifer-hosted microbial assemblages are sufficiently diverse to adapt to a range of redox conditions, selecting favorable environment-dictated bacterial community membership across a 60°C temperature range within relatively short biological temporal scales (<1 year).

In the Hole U1301A incubation, similar taxa prevailed across mineral substrates (Table 1), suggesting that mineral composition was not a primary driver of microbial community structure in this 1-year experiment. The close physical association of the minerals in serially connected cassettes within the FLOCS experiment may have allowed crossover between treatments, thus allowing overprinting and blending between treatments that could not be resolved with this approach. Regardless, the dominant, primarily Epsilonproteobacteria taxa may have had an adaptive strategy for harnessing reduced elements from either the mineral surfaces or dissolved in the crustal fluids, despite the mineral composition.

### The potential for S and Fe oxidation by subsurface microbial communities under changing environmental conditions

The most prevalent taxa observed in the wellhead colonization experiments were the Epsilonproteobacteria, which are capable of carbon fixation via sulfide oxidation (Wirsen et al., 2002) and are known to be the predominant organisms found in hydrothermal environments (Nakagawa et al., 2005; Huber et al., 2007). Epsilonproteobacteria are also detected on seafloor exposed massive sulfides (Sylvan et al., 2012; Toner et al., 2013). The most prevalent species observed in the FLOCS was OTU4 (101 of 370 clones), classified as *Arcobacter*, but with only 95% sequence similarity to other known *Arcobacter* species (Table 1, Figure 4). This OTU was primarily found on pyrite, goethite, hematite and the glass wool + pyrrhotite, but also on basalt (Table 1). Other *Arcobacter*-related OTUs were also observed (Table 1, Figure 4). *Arcobacter sp*. are capable of sulfide oxidation (Wirsen et al., 2002) and it is possible that these organisms utilize leaching sulfur species from pyrite and pyrrhotite with oxygen present within the FLOCS chambers. We hypothesize, based on the high prevalence of 16S rRNA genes classified as Epsilonproteobacteria, that sulfur oxidation is a key metabolic pathway supporting mineral-microbe communities under cool and oxic conditions. Given that such Epsilonproteobacteria are known to use the reverse tricarboxylic acid (rTCA) cycle for carbon fixation (Hugler et al., 2005), it is possible that autotrophic metabolisms are also supported in this system by this cohort.

Under the cool and presumably oxic conditions of the wellhead FLOCS experiment, oxidation of sulfur and iron are thermodynamically favorable (Orcutt et al., 2011a). Only one 16S rRNA gene sequence from this study was associated with known iron oxidizing bacteria of the Zetaproteobacteria (Table 1), suggesting that iron oxidation may not have been a prominent metabolic process in the wellhead FLOCS environment at the time of collection. The low abundance of known iron oxidizing bacteria in the wellhead FLOCS clone libraries is consistent with previous studies from other iron-oxidizing mineral systems (Thorseth et al., 2001; Edwards et al., 2003; Santelli et al., 2008; Sylvan et al., 2012). This suggests either that (i) known iron oxidizers (i.e., Zetaproteobacteria) make up a small fraction of the microbial community; (ii) that multiple displacement amplification and/or primers used to construct clone libraries are biased against the proportional representation of known iron oxidizers; (iii) that iron oxidation may only be significant under a brief time window early in the experiment, leaving only fossil biominerals by the time of collection, as suggested previously (Orcutt et al., 2011a), or (iv) that iron oxidation was mediated by other unknown groups within the microbial community. Importantly, the observation of any known iron oxidizing bacteria or biomineral markers of their activity is again reflective of the presence of oxygen in the wellhead FLOCS experiments, as these microorganisms are not known from anoxic environments (Emerson et al., 2010).

We stress that observations regarding S and Fe cycling in this study are only applicable to our incubation conditions (i.e., the oxic and cold conditions of the FLOCS at the wellhead environment) and thus do not necessarily reflect subseafloor microbial activities at this site; however, our results show an adaptive response to changing thermal and redox regimes, and suggest an effective adaptation capacity potential for the colonization of mineral substrates by subseafloor crustal fluid biota.

## Conclusions and recommendations

As a tool for examining mineral-microbe interactions in the warm (64°C) and anoxic crustal subsurface, deployment of mineral colonization experiments at the wellhead of crustal borehole observatories must be configured to mimic conditions at depth. This can be conducted in multiple ways; however the easiest means is to place such experiments within the open borehole of overpressured observatories from which hydrothermal fluids vent freely (Fisher et al., 2011). Our approach has value for studying cooler and oxic subsurface crustal communities, which are widespread (Edwards et al., 2012a,b; Orcutt et al., 2013b). Results of our experiment illustrate that low abundance members of Juan de Fuca crustal fluids, particularly low abundance crustal taxa classified as Epsilonproteobacteria, proliferate under changing environmental conditions and become dominant members of the microbial community on substrates incubated for a year in the cool and oxic bottom water-hosted system at the CORK wellhead. Different mineral surfaces inoculated with Juan de Fuca crustal fluids do not induce growth of specific communities within a 1-year timeframe. Redox conditions (oxic/anoxic) and temperature (4/64°C) dictate substrate colonization and community succession, and are proposed as major ecological drivers in environments where crustal fluids interact with minerals. Thus, to further the quest of explaining the establishment, adaptation, and activities of microbial life in the marine deep biosphere, future FLOCS experiments can be designed to elucidate mechanisms and/or rate of specific mineral-microbial interactions under a range of thermal and chemical conditions.

## Author contributions

All authors contributed to the design of the experiment. KE and SH deployed the experiment; KE, GR, BO, SH, GW recovered the experiment. JB, GR, and AH analyzed samples, and all authors contributed to data analysis. JB, GR, KE, AH, and BO wrote the paper with input from all authors.

### Conflict of interest statement

The authors declare that the research was conducted in the absence of any commercial or financial relationships that could be construed as a potential conflict of interest.

## Abbreviations

CORK: Circulation Obviation Retrofit Kit (a borehole observatory)
FLOCS: Flow-through Osmo Colonization System (a flow-through chamber for examining microbe-mineral interactions where fluid is pulled through by the action of an attached OsmoSampler osmotic pump)
IODP: Integrated Ocean Drilling Program
mbsf: meters below seafloor.
